# How Many Urine Samples Are Needed to Accurately Assess Exposure to Non-Persistent Chemicals? The Biomarker Reliability Assessment Tool (BRAT) for Scientists, Research Sponsors, and Risk Managers

**DOI:** 10.3390/ijerph17239102

**Published:** 2020-12-06

**Authors:** Marc-André Verner, Hassan Salame, Conrad Housand, Linda S. Birnbaum, Maryse F. Bouchard, Jonathan Chevrier, Lesa L. Aylward, Daniel Q. Naiman, Judy S. LaKind

**Affiliations:** 1 Department of Occupational and Environmental Health, School of Public Health, Université de Montréal, Montreal, QC H3T 1A8, Canada; hasan_salame@hotmail.com (H.S.); maryse.bouchard@umontreal.ca (M.F.B.); 2 Centre de Recherche en Santé Publique, Université de Montréal et CIUSSS du Centre-Sud-de-l’Île-de-Montréal, Montreal, QC H3C 3J7, Canada; 3 Independent Consultant, Winter Springs, FL 32708, USA; cjhousand@cfl.rr.com; 4 Scientist Emeritus and Former Director, National Institute of Environmental Health Sciences and National Toxicology Program, Research Triangle Park, NC 27514, USA; birnbaum.tox@outlook.com; 5 CHU Sainte-Justine Research Centre Mother and Child University Hospital Center, Montreal, QC H3T 1C5, Canada; 6 Department of Epidemiology, Biostatistics and Occupational Health, McGill University Faculty of Medicine, Montreal, QC H3A 1A3, Canada; jonathan.chevrier@mcgill.ca; 7 Summit Toxicology, LLP, Falls Church, VA 22044, USA; laylward@summittoxicology.com; 8 Queensland Alliance for Environmental Health Sciences, University of Queensland, Brisbane, QLD 4102, Australia; 9 Department of Applied Mathematics and Statistics, The Johns Hopkins University, Baltimore, MD 21218, USA; daniel.naiman@jhu.edu; 10 LaKind Associates, LLC, Catonsville, MD 21228, USA; lakindassoc@gmail.com; 11 Department of Epidemiology and Public Health, University of Maryland School of Medicine, Baltimore, MD 21201, USA

**Keywords:** biomonitoring, non-persistent chemicals, exposure assessment, within- and between-person variability, environmental epidemiology, urine sampling, pharmacokinetic modeling, exposure misclassification

## Abstract

In epidemiologic and exposure research, biomonitoring is often used as the basis for assessing human exposure to environmental chemicals. Studies frequently rely on a single urinary measurement per participant to assess exposure to non-persistent chemicals. However, there is a growing consensus that single urine samples may be insufficient for adequately estimating exposure. The question then arises: how many samples would be needed for optimal characterization of exposure? To help researchers answer this question, we developed a tool called the Biomarker Reliability Assessment Tool (BRAT). The BRAT is based on pharmacokinetic modeling simulations, is freely available, and is designed to help researchers determine the approximate number of urine samples needed to optimize exposure assessment. The BRAT performs Monte Carlo simulations of exposure to estimate internal levels and resulting urinary concentrations in individuals from a population based on user-specified inputs (e.g., biological half-life, within- and between-person variability in exposure). The BRAT evaluates—through linear regression and quantile classification—the precision/accuracy of the estimation of internal levels depending on the number of urine samples. This tool should guide researchers towards more robust biomonitoring and improved exposure classification in epidemiologic and exposure research, which should in turn improve the translation of that research into decision-making.

## 1. Introduction

In epidemiologic and exposure research, biomonitoring (i.e., measuring the concentration of specific chemicals or their metabolites in biological samples) is often used as the basis for assessing human exposure to environmental chemicals. A positive attribute of biomonitoring is that the measurements provide an estimate of the combined absorbed dose of chemicals from all routes of exposure, including oral, dermal, and inhalation [1]. Urine is the most widely used biological matrix for measuring exposure to non-persistent chemicals such as bisphenols, phthalates, triclosan, organophosphate pesticides, and flame retardants, and is also the matrix of choice because concentrations are typically higher in urine compared to other matrices and so are more readily measurable [2,3]. Urine sampling also has the advantage of being a non-invasive procedure amenable to field studies and only requires minimally trained staff, unlike blood sampling that must be performed by a phlebotomist. 

Epidemiologic studies often rely on a single urinary measurement per participant to evaluate associations between exposure to non-persistent chemicals and health outcomes [4]. For instance, in the case of bisphenol A (BPA), 41 out of 45 studies reviewed by LaKind et al. [5] used measurements from a single spot urine sample to assess individual exposures and to link those exposures to health outcomes. Likewise, exposure studies frequently measure non-persistent chemicals in a single spot urine sample per participant to investigate the determinants of exposure (e.g., age, consumption of certain food items). However, the use of a single spot measurement can result in exposure misclassification due to the short physiologic half-lives of these chemicals, inconsistency in time of sampling in relation to most recent exposures, and temporal variations in the exposures themselves [6]. The reliability of one spot measurement as an indicator of exposure has been evaluated in many studies, most often through the calculation of intraclass correlation coefficients (ICCs) [7,8,9,10]. These coefficients are calculated as the ratio of inter-individual variance to the total variance using data from serial sampling of the chemical over several hours, days, or weeks. A review of recent literature has found that approximately 60% of ICC values reported for non-persistent chemicals fall under 0.4, a value corresponding to poor reliability for a single measurement [4]. Even for chemicals with higher ICCs, a certain degree of exposure misclassification can still occur [6,7]. 

There is a growing consensus that a single urine sample may not be sufficient to adequately assess exposure to non-persistent chemicals in many circumstances. Consequently, epidemiologic studies using single spot samples are more poorly rated in risk assessment documents [11] and may have more limited impacts on the development of health-based guidance than studies using multiple samples. But the question then arises, how many samples are needed for an accurate characterization of exposure to a given chemical, depending on the sampling conditions? Previous work has been done towards estimating the number of samples needed using different statistical approaches including the Spearman-Brown equation [12] and equations based on the coefficient of variation of repeated urinary concentrations [13]. However, there are issues around the use of published ICC values for determining the number of urine samples needed for exposure assessment [7]. For instance, this approach is limited by the specificity of ICCs to the sampling scheme, the relative inter- and intra-individual variability inherent to the study population, and the method used to standardize concentrations to account for urine dilution. For example, a study of a sample population regularly exposed to a chemical and for which sampling was conducted over a short time period (e.g., samples collected at the same time for each individual and for which exposure occurred at the same time daily over that sampling period) would yield an ICC that may not apply to another population with longer and more variable exposure and sampling patterns. Statistical simulations have also been used to determine how many samples are required to minimize bias in epidemiologic studies [14], but these calculations were also based on ICCs, which can vary substantially from one study to another depending on the population and chemical. 

Ideally, the number of samples for both epidemiology and exposure research should be determined based on information on population- and chemical-specific parameters including biological half-life, exposure and sampling patterns, and intra-individual and inter-individual variability in exposure levels between exposure events. Researchers designing studies and reviewing grant proposals and manuscripts and those using epidemiology and exposure research for public-health decision-making would benefit from an easy-to-use tool that could guide them towards an improved understanding of the adequate number of urine samples to achieve reliable exposure estimates.

In this paper, we describe a tool that we developed called the Biomarker Reliability Assessment Tool (BRAT). BRAT is based on pharmacokinetic modeling simulations and is designed to assist researchers in estimating the number of urine samples needed to reliably capture human exposures during toxicologically relevant periods.

## 2. Materials and Methods 

### 2.1. Overview

**Figure 1:**
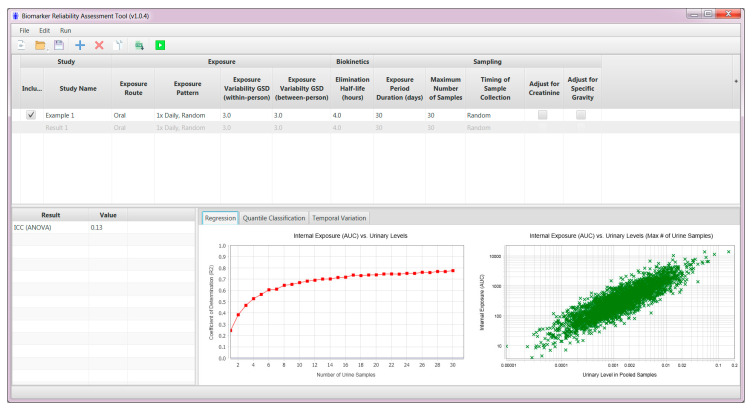
Biomarker Reliability Assessment Tool (BRAT) user interface.

BRAT was developed for broad use and a beta version is freely available online at: https://www.magnoliasci.com/brat. The BRAT user interface is shown in Figure 1. 

The BRAT software uses a simple pharmacokinetic model to simulate exposure, internal levels, and resulting urinary concentrations in individuals from a population. The output is based on user-specified inputs (e.g., biological half-life, within- and between-person variability in exposure). Based on internal concentrations (a toxicologically relevant exposure metric) and urinary concentrations in the simulated population, the tool evaluates—through linear regression and quantile classification—the accuracy of the estimation of internal levels depending on the number of urine samples. The graphics generated by BRAT provide a visual basis to optimize the number of urine samples to be collected and optimal timing of sample collection for exposure assessment.

### 2.2. System Requirements

The BRAT software is a desktop application which runs on 64-bit Windows 10 and is distributed as a standard Windows installer package. BRAT is a self-contained application in that it does not require installation of any additional third-party tools to provide computational/statistical capabilities (e.g., MATLAB or R). The simulation engine and ordinary differential equations (ODE) solver are provided by the Magnolia modeling and simulation runtime library, which is fully embedded in BRAT. Recommended computer specifications to use BRAT are at least 8 Gb of RAM, a quad-core processor, and 500 Mb of free disk space. 

### 2.3. User Interface—Model Inputs

The user interface allows users to specify several chemical- and study-specific parameters as shown in Table 1. In addition, users can change the number of individuals (default set to 3000), the accumulation period (i.e., a period of exposure prior to urine sampling—default set to 7 times the biological half-life, with a minimum of 14 days), variability in biological half-life (default set to a GSD of 1.3 based on Spaan [15]), and oral absorption half-life (default set to 0.4 hour based on Poulin [16]). These changes are made in the Settings under the File dropdown menu. Whenever chemical-specific data are available, default values should be replaced.

### 2.4. Pharmacokinetic Model Simulations 

The software uses a pharmacokinetic model and Monte Carlo simulations to simulate exposure and urinary elimination in a population of individuals. For each simulated individual, a geometric mean dose is sampled from a log-normal distribution with a geometric mean of 1 and the between-person GSD specified by the user. The dose at each exposure event is sampled from a log-normal distribution based on that geometric mean and user-specified within-person GSD.

BRAT uses data on urine volume, time of urination events, creatinine urinary concentration, and urine specific gravity from eight individuals (four men and four women) who provided urine over six consecutive days [17]. For each simulated individual, one of the eight participants is randomly selected, and the urination schedule for each day of the simulation is randomly selected from the six days of data collection for the selected individual.

**Figure 2:**
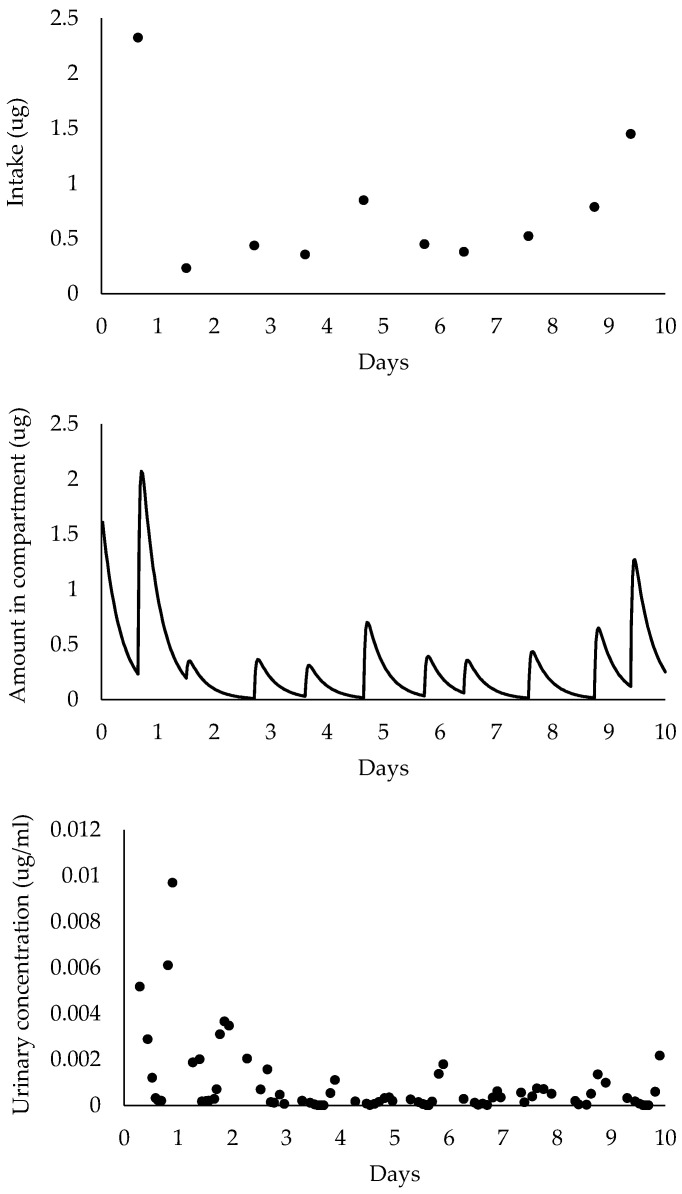
Simulated intake, amount in central compartment, and urinary concentrations over the course of 10 days for one individual exposed to a chemical with a half-life of 4 hours once a day at random times. Of note, model inputs are those presented in Figure 1.

BRAT generates complete profiles of external exposure (intake), internal dosimetry (amount of chemical in the central compartment of the pharmacokinetic model), and urinary chemical concentrations for each individual throughout the simulation time (see Figure 2). The area under the internal concentration vs. time curve (AUC) is considered to represent the most toxicologically relevant metric for potential systemic effects. If a certain window of susceptibility is hypothesized, for example the first trimester of pregnancy, the toxicologically relevant period could be three months. Of note, the tool was not developed to address lifetime exposures in the context of chronic or latent disease (e.g., cancer) as exposure levels may change across lifestages. Where multiple periods are hypothesized to be toxicologically relevant, results from the BRAT could be used to estimate the number of urine samples to collect during each time window. From all simulated urinary concentrations for an individual, urine samples are “collected” based on user-specified timing of collection and number of samples.

### 2.5. Model Outputs

After a BRAT run is completed, results from simulations are presented in the bottom half of the user interface under three tabs (regression, quantile classification, temporal variation) and an ICC calculated using the ANOVA method (see example in Figure 1). The ability to represent overall “true internal dosimetry” (i.e., the area under the curve [AUC]) from pooled urine samples is assessed through different statistical approaches. 

#### 2.5.1. Regression Tab

**Figure 3:**
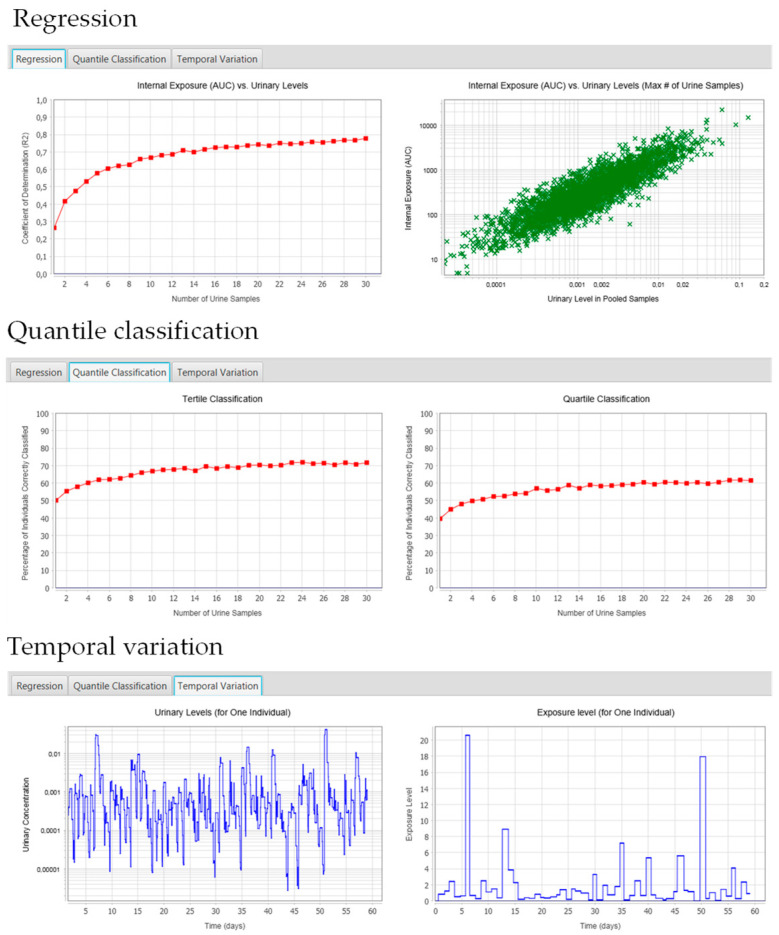
Results presented in the BRAT interface after a run is completed. The figure presents results from the regression tab, the quantile classification tab, and the temporal variation tab.

Two plots are generated under the regression tab (see Figure 3). The plot on the left (internal exposure [AUC] vs. urinary levels) shows the coefficient of determination (R^2^) for the regression of log-transformed areas under the curve (AUCs) and mean urinary concentrations. The first point on the left represents the R^2^ for the regression of AUC and the concentration in one urine sample. The other points represent the R^2^ for the regression between AUCs and the concentration in equal-volume pools of urine samples (mean concentration). For example, the R^2^ for the second point is for the regression of AUC and the mean urinary concentration from two urine samples. The R^2^ generally increases as the number of collected urine samples increases. Although the plot does not give the user the “correct” or “optimal” number of urine samples for a given study, researchers may use it to balance the costs/logistics associated with additional sampling against the increase in R^2^. In the example presented in Figure 3, collecting more than 10 urine samples only minimally improves R^2^. The scatter plot on the right (internal exposure [AUC] vs. urinary levels [Maximum # of urine samples]) shows the AUCs and mean urinary concentrations based on the maximum number of samples you entered as an input parameter in BRAT. In Figure 3, this plot shows the AUC versus the mean urinary concentration in 30 samples for each simulated individual. 

#### 2.5.2. Quantile Classification

Two plots are generated under the quantile classification tab (see Figure 3). These plots show the percentage of individuals correctly classified in exposure tertiles/quartiles based on urinary measurements vs. the “true internal exposure” (AUC). The first point on the left represents the percentage of individuals correctly classified based on one urine sample. The following points represent the percentage of individuals correctly classified based on equal-volume pools of urine samples (mean concentration). In the example shown in Figure 3, the percentage of individuals correctly classified increases as you increase the number of samples, but the increase plateaus above ~10 samples.

#### 2.5.3. Temporal Variation Tab

Two plots are generated under the temporal variation tab (see Figure 3). The plot on the left (urinary levels for one individual) represents the variation in urinary concentrations over the exposure period for one of the simulated individuals. The variability in urinary concentrations over time can help scientists evaluate the magnitude of temporal changes in urinary concentrations and compare with published temporal profiles of urinary concentrations. The plot on the right (intake) represents the variation in chemical intake for each exposure event over the exposure period for one of the simulated individuals.

## 3. Discussion

We developed the BRAT to assist in the determination of the optimal number of urine samples to collect to accurately characterize exposure. Researchers as well as manuscript and grant reviewers could use this tool to assess the reliability of exposure estimates in different study contexts. Of course, the optimal number of samples estimated with the BRAT analysis will inevitably have to be balanced against logistical and financial limitations, as well as acceptability for study participants. Under some scenarios, it may not be possible to collect the optimal number of samples. It should be noted that the BRAT assumes that urine samples are pooled (equal-volume pools) prior to laboratory determination of chemical concentrations, so the added costs of repeated sampling are mainly related to sample collection.

Epidemiologic studies have been used extensively for the hazard identification component of risk assessment, but fewer have been considered sufficiently robust to be used directly in the development of guidance values (e.g., used in dose-response assessment) [18,19]. Because of what are often considered inconsistent results across epidemiologic studies as well as other challenges associated with observational studies, agencies often base their guidance values on animal studies despite uncertainty in extrapolating results to human populations. Exposure misclassification resulting from an insufficient number of biological samples has been described for multiple chemicals, such as bisphenols, pyrethroids, and triclosan [7,10,20]. This misclassification is likely to contribute to discrepancies across studies, especially for chemicals with a short biological half-life and sporadic exposure events. Future studies using the BRAT to determine the adequate number of samples, and collecting those samples for a reliable exposure assessment, could generate more reproducible results. There are several challenges in using epidemiologic data in regulatory risk assessment, but as human data provide unique information beyond what can be gleaned from traditional toxicology-based risk assessments, further efforts should be devoted to overcome these challenges. More reliable, robust exposure assessment is a key aspect of achieving this goal. 

The BRAT should be considered living software. While the software is functional for certain exposure scenarios, not all scenarios of interest are covered by this first version of the tool. It is anticipated that modifications to BRAT will be made in the future to address current limitations. For example, the tool uses urine data (i.e., volume, collection timing, creatinine, specific gravity) from eight adults (four females and four males) over six consecutive days to calculate urinary concentrations. Therefore, the BRAT may not generate results that are applicable to infants, toddlers, and children. With that being said, the variability in 24-hour urine volume, voiding frequency, and creatinine concentration was similar to that reported in reference populations, suggesting data from these eight individuals may be sufficient to represent variability in adults from the population [21,22]. As new data are made available to us, they could be incorporated into BRAT updates. Further, the tool does not currently address the dermal or inhalation exposure routes, or multiple exposure routes. It also only accommodates equal-volume pooling of urine samples; other pooling approaches (e.g., based on creatinine) are not included. Finally, except for whole day volume weighted concentrations, a maximum of one biomonitoring sample (random, first morning/evening void) per day is currently allowed as a model input. Despite these shortcomings, we believe the BRAT is a promising approach to improving exposure assessment based on biomonitoring.

## 4. Conclusions

Currently, epidemiologic and exposure studies that use biomonitoring to characterize exposure to non-persistent chemicals often rely on one or few urine samples. We now understand that this is generally insufficient for properly characterizing exposure. However, to date, there has not been a readily available tool for estimating the number of samples needed for a robust exposure assessment. The BRAT was designed to address this issue. As shown in the example in this paper, a small increase in the number of urine samples (e.g., from one to four) could substantially increase the precision of exposure estimates.

Our goal is to continually improve the BRAT to include additional functionalities to address other exposure scenarios. The ultimate goal is to obtain more robust biomonitoring information that will improve exposure classification in epidemiologic research; this, in turn, will enhance the translation of that research for decision-making [23].

## Figures and Tables

**Table 1:** Biomarker Reliability Assessment Tool (BRAT) parameters and inputs.

Parameter	Input
Exposure route	At present, the tool accommodates the oral route of exposure
Exposure pattern	Once a day at a random time Once a day during the evening Twice a day at random times Twice a day (morning and afternoon) Three times a day at random times Three times a day (morning, afternoon and evening)
Within-person variability in exposure levels	Geometric standard deviation for a distribution of within-person exposure levels (including all exposure events)
Between-person variability in exposure levels	Geometric standard deviation for a distribution of geometric mean exposure levels in individuals
Biological half-life	Biological half-life in hours
Exposure period of interest	Duration of the toxicologically relevant period of exposure in days
Maximum number of samples that can be collected	The maximum number of urine samples that can realistically be collected from participants
Timing of sample collection	Random First morning void First evening void Whole day volume weighted
Standardization for urine dilution	No standardization Standardization for creatinine Standardization for specific gravity

## References

[1] Aylward L.L., Hays S.M., Smolders R., Koch H.M., Cocker J., Jones K., Warren N., Levy L., Bevan R. (2014). Sources of variability in biomarker concentrations. J. Toxicol. Environ. Health B Crit. Rev..

[2] Barr D.B., Wang R.Y., Needham L.L. (2005). Biologic monitoring of exposure to environmental chemicals throughout the life stages: Requirements and issues for consideration for the National Children’s Study. Environ. Health Perspect..

[3] Calafat A.M., Longnecker M.P., Koch H.M., Swan S.H., Hauser R., Goldman L.R., Lanphear B.P., Rudel R.A., Engel S.M., Teitelbaum S.L. (2005). Optimal Exposure Biomarkers for Nonpersistent Chemicals in Environmental Epidemiology. Environ. Health Perspect..

[4] LaKind J.S., Idri F., Naiman D.Q., Verner M.A. (2019). Biomonitoring and Nonpersistent Chemicals-Understanding and Addressing Variability and Exposure Misclassification. Curr. Environ. Health Rep..

[5] LaKind J.S., Goodman M., Mattison D.R. (2014). Bisphenol A and indicators of obesity, glucose metabolism/type 2 diabetes and cardiovascular disease: A systematic review of epidemiologic research. Crit. Rev. Toxicol..

[6] Goodman M., Lakind J.S., Mattison D.R. (2014). Do phthalates act as obesogens in humans? A systematic review of the epidemiological literature. Crit. Rev. Toxicol..

[7] Goodman M., Naiman D.Q., LaKind J.S. (2018). Systematic review of the literature on triclosan and health outcomes in humans. Crit. Rev. Toxicol..

[8] Bertelsen R.J., Engel S.M., Jusko T.A., Calafat A.M., Hoppin J.A., London S.J., Eggesbø M., Aase H., Zeiner P., Reichborn-Kjennerud T. (2014). Reliability of triclosan measures in repeated urine samples from Norwegian pregnant women. J. Expo. Sci. Environ. Epidemiol..

[9] Vernet C., Philippat C., Calafat A.M., Ye X., Lyon-Caen S., Siroux V., Schisterman E.F., Slama R. (2018). Within-Day, Between-Day, and Between-Week Variability of Urinary Concentrations of Phenol Biomarkers in Pregnant Women. Environ. Health Perspect..

[10] Morgan M.K., Sobus J.R., Barr D.B., Croghan C.W., Chen F.L., Walker R., Alston L., Andersen E., Clifton M.S. (2016). Temporal variability of pyrethroid metabolite levels in bedtime, morning, and 24-h urine samples for 50 adults in North Carolina. Environ. Res..

[11] US Environmental Protection Agency (2018). Application of Systematic Review in TSCA Evaluations. EPA Document# 740-P1-8001.

[12] Casas M., Basagaña X., Sakhi A.K., Haug L.S., Philippat C., Granum B., Manzano-Salgado C.B., Brochot C., Zeman F., de Bont J. (2018). Variability of urinary concentrations of non-persistent chemicals in pregnant women and school-aged children. Environ. Int..

[13] Li A.J., Martinez-Moral M.P., Kannan K. (2020). Variability in urinary neonicotinoid concentrations in single-spot and first-morning void and its association with oxidative stress markers. Environ. Int..

[14] Perrier F., Giorgis-Allemand L., Slama R., Philippat C. (2016). Within-subject Pooling of Biological Samples to Reduce Exposure Misclassification in Biomarker-based Studies. Epidemiology.

[15] Spaan S., Fransman W., Warren N., Cotton R., Cocker J., Tielemans E. (2010). Variability of biomarkers in volunteer studies: The biological component. Toxicol. Lett..

[16] Poulin P., Jones R.D., Jones H.M., Gibson C.R., Rowland M., Chien J.Y., Ring B.J., Adkison K.K., Ku M.S., He H. (2011). PHRMA CPCDC initiative on predictive models of human pharmacokinetics, part 5: Prediction of plasma concentration-time profiles in human by using the physiologically-based pharmacokinetic modeling approach. J. Pharm. Sci..

[17] Smolders R., Koch H.M., Moos R.K., Cocker J., Jones K., Warren N., Levy L., Bevan R., Hays S.M., Aylward L.L. (2014). Inter- and intra-individual variation in urinary biomarker concentrations over a 6-day sampling period. Part 1: Metals. Toxicol. Lett..

[18] Persad A.S., Cooper G.S. (2008). Use of epidemiologic data in Integrated Risk Information System (IRIS) assessments. Toxicol. Appl. Pharmacol..

[19] Ockleford C., Adriaanse P., Berny P., Brock T., Duquesne S., Grilli S., Hougaard S., Klein M., Kuhl T., EFSA PPR Panel [EFSA Panel on Plant Protection Products and their Residues] (2017). Scientific Opinion of the PPR Panel on the follow-up of the findings of the External Scientific Report ‘Literature review of epidemiological studies linking exposure to pesticides and health effects’. EFSA J..

[20] Faÿs F., Palazzi P., Hardy E.M., Schaeffer C., Phillipat C., Zeimet E., Vaillant M., Beausoleil C., Rousselle C., Slama R. (2020). Is there an optimal sampling time and number of samples for assessing exposure to fast elimination endocrine disruptors with urinary biomarkers?. Sci. Total Environ..

[21] Van Haarst E.P., Heldeweg E.A., Newling D.W., Schlatmann T.J. (2004). The 24-h frequency-volume chart in adults reporting no voiding complaints: Defining reference values and analysing variables. BJU Int..

[22] Barr D.B., Wilder L.C., Caudill S.P., Gonzalez A.J., Needham L.L., Pirkle J.L. (2005). Urinary creatinine concentrations in the U.S. population: Implications for urinary biologic monitoring measurements. Environ. Health Perspect..

[23] LaKind J.S., Burns C.J., Erickson H., Graham S.E., Jenkins S., Johnson G.T. (2020). Bridging the Epidemiology Risk Assessment Gap: An NO_2_ Case Study of the Matrix. Glob. Epidemiol..

